# The global burden of lead exposure-related ischemic stroke: based on Bayesian age-period-cohort analysis

**DOI:** 10.3389/fpubh.2025.1608129

**Published:** 2025-07-16

**Authors:** Xiaofang Chen, Lihua Zhao, Xiaxia Wu, Dan Chen, Mingya Yao

**Affiliations:** Department of Neurosurgery, The First Affiliated Hospital of Wenzhou Medical University, Wenzhou, China

**Keywords:** disease burden, lead exposure, ischemic stroke, trend, prediction

## Abstract

**Introduction:**

The global burden of lead exposure-related ischemic stroke poses a significant concern for public health, and this study aimed to comprehensively analyze the current situation, the change patterns, and forecast potential future trends associated with this critical issue.

**Methods:**

The Global Burden of Disease (GBD) 2019 dataset was analyzed to assess the burden of lead exposure-related ischemic stroke. Comparison of the 2019 disease burden was conducted across age, gender, Sociodemographic Index (SDI) regions, and GBD regions. Linear regression models were used to calculate the estimated annual percentage change (EAPC) values, exploring trends from 1990 to 2019. Cluster analysis identified patterns of changing disease burden across GBD regions. Bayesian age-period-cohort (BAPC) analysis was performed to predict future burden trends.

**Results:**

In 2019, lead exposure-related ischemic stroke caused a significant disease burden, with males and middle-aged/older adults disproportionately affected. The highest burden was observed in middle/low-middle SDI regions. From 1990 to 2019, the age-standardized deaths rate (ASDR) of deaths and disability-adjusted life-years (DALYs) exhibited a rise-and-fall pattern, with absolute cases increasing. Males consistently carried a heavier burden, and age groups exhibited variable patterns but generally followed the overall trend. High SDI regions saw a decline in burden, whereas other regions mirrored the global pattern. Clustering analysis revealed region-specific variations. The BAPC model predicts differing global trends in lead exposure-related ischemic stroke burden genders, with females expecting stable cases but decreasing rates, while males anticipate declines in all indicators over the next 11 years.

**Conclusion:**

The global burden of lead exposure-related ischemic stroke is a pressing issue that requires urgent attention. Our findings underscore the need for enhanced surveillance, prevention, and treatment strategies to mitigate this burden.

## Introduction

Among numerous cardiovascular diseases, stroke is one of the most serious causes of death and severe disability worldwide (1). In 2019, 6.55 million people worldwide died from stroke, and 143 million in disability adjusted life years died from stroke (2). Ischemic stroke is the most common type, accounting for 70%−80% of all stroke cases, while hemorrhagic stroke affects ~10%−20% of stroke patients (3, 4). Ischemic stroke is a serious disease, which has been considered as the main cause of global incidence rate and mortality for a long time (5, 6), posing a significant burden on individuals and families. Its impact is profound, often resulting in long-term disabilities and substantial healthcare costs with profound societal and economic implications (7–9). There is a positive association between lead exposure and coronary artery disease, stroke mortality, and peripheral arterial disease, as identified in studies of general populations (10). The National Health and Nutrition Examination Survey cohort study in the US demonstrated a dose-response relationship between blood lead level and stroke mortality (11). Lead exposure may increase the risk of ischemic stroke through pathways such as damaging vascular endothelium, promoting hypertension and arteriosclerosis, inducing inflammation and oxidative stress (12). A thorough evaluation of the ischemic stroke burden linked to lead exposure will assist in creating effective prevention strategies and minimizing the stroke burden.

Lead, a ubiquitous environmental toxin, is a persistent and widespread contaminant that poses a significant threat to human health (13, 14). Lead can be encountered through dust, old lead paints, drinking water, or food (15–17). From the 1920s to the 1980s, tetraethyl lead was added to gasoline to prevent engine knocking, leading to global atmospheric lead pollution (18). This period coincided with rising cardiovascular disease rates, including ischemic stroke, though early research focused on occupational exposure (e.g., miners, battery workers) rather than environmental impacts. Lead was also used in paints, plumbing, and food containers, leading to chronic low-dose exposure in the general population. For example, children's blood lead levels (BLLs) in the U.S. peaked at 12 μg/dl in the 1970s (compared to <1 μg/dl today) (18). Before being banned, leaded gasoline was a significant cause of lead poisoning, as noted by historical sources (19). Despite the Chinese government's prohibition of leaded gasoline in 2000, the rapidly growing lead-acid battery sector, the rise of e-waste recycling, and metal smelting have emerged as significant human-made sources of lead pollution (20). In nations that have stopped using leaded gasoline, there has been a quick reduction in average BLLs (21). Some studies say that even at low levels, lead exposure will increase the incidence rate of hypertension, stroke and other cardiovascular diseases (22). In addition, a study suggests that groups with elevated bone lead levels have a higher risk of future ischemic heart disease (IHD), as lead exposure can damage the inner walls of blood vessels, leading to inflammation and plaque formation, resulting in narrowing of blood vessels (23). In a prospective study of the population cohort in 2017, it was found that left ventricular systolic function significantly decreased due to environmental lead exposure (24). However, despite this well-established link, the specific burden of ischemic stroke attributed to lead exposure remains less understood. This knowledge gap is particularly concerning given the potential for lead exposure to contribute significantly to the already substantial global burden of ischemic stroke.

The Global Burden of Disease (GBD) Study has been instrumental in offering crucial insights into the scale and distribution of disease burdens worldwide. These studies have been essential in pinpointing key areas for intervention and elucidating the trends and patterns of disease occurrence over time (2, 25). Despite the thoroughness of the GBD 2019 study, no prior research has offered an in-depth analysis of the burden of ischemic stroke specifically attributed to lead exposure using the GBD 2019 database.

This study seeks to fill a critical knowledge gap by analyzing data from the Global Burden of Disease 2019 Study. Our goal is to gain a deeper understanding of the global burden of lead-related ischemic stroke and to examine the trends in disease burden over the past 30 years. Additionally, we employ Bayesian age-period-cohort (BAPC) analysis to predict future trends in the incidence of this condition. These insights are essential for devising effective strategies to mitigate the impact of lead exposure on ischemic stroke and to improve global health outcomes. By bridging this knowledge gap, we can contribute to the global effort to reduce the burden of ischemic stroke and enhance the overall health and quality of life for people and communities around the globe.

## Methods

### Data sources

In this study, we employed the GBD 2019 dataset as our primary source of information, accessible at https://vizhub.healthdata.org/gbd-compare/. This extensive dataset provides estimations of ischemic stroke deaths and disability-adjusted life-years (DALYs) linked to lead exposure, encompassing global, regional, and national scopes from 1990 to 2019. Leveraging this comprehensive dataset, we conducted a rigorous analysis to assess the burden of ischemic stroke attributed to lead exposure. Annual data on the global burden of stroke attributable to lead exposure were retrieved from the Institute for Health Metrics and Evaluation's online query tool (http://ghdx.healthdata.org/gbd-results-tool), adhering to the criteria of (1) geographical scope included global, regional, and national levels, with data spanning the years 1990 to 2019, and the risk factor specified as “lead exposure”; (2) disease outcomes focused on “stroke,” including its subtypes “ischemic stroke”; and (3) health impact measures were limited to “Deaths” and “Disability-Adjusted Life Years (DALYs).” The burden of disease was quantified using the number of cases and age-standardized deaths rate (ASDR) per 100,000 population as our key indicator. This allowed us to compare and contrast the impact of lead exposure across different age groups, sexes, and geographical regions. The general methodology employed for GBD 2019 has been outlined in previous publications, ensuring a clear and logical framework for our analysis (26).

To ensure the accuracy and reliability of our findings, we collected both age-standardized and all-age data across all age groups and both sexes. The data of the GBD database were sourced from a range of reliable sources, including vital statistics, published literature, survey and surveillance data, as well as health insurance claims (27, 28). Data from various sources were combined to model and estimate the burden of disease indicators. To ensure consistency and adjust for methodological differences, we used DisMod-MR 2.1, a Bayesian meta-regression tool, to model non-fatal outcomes. This tool allowed for standardized calculations across different estimates. DisMod-MR generated comprehensive age/sex/region/year-specific estimates for non-fatal burden of disease indicators (29).

The GBD study determined DALYs by combining years of life lost (YLLs) and years lived with disability (YLDs) for each age group, sex, and year (2). Additionally, the Sociodemographic Index (SDI), which accounts for income per capita, average educational attainment, and fertility rates, was used in this database. For more details, refer to previous studies (30, 31). To model mortality rates, the GBD cause of death ensemble model was employed, offering a comprehensive framework to evaluate the global burden of lead exposure-related ischemic stroke (32).

### Statistics analysis

This study employed a three-step analysis to thoroughly evaluate the burden of ischemic stroke attributed to lead exposure. First, a descriptive epidemiological analysis was conducted to examine the global and subtype distributions of the ischemic stroke burden associated with lead exposure, considering factors such as age, gender, SDI regions, and GBD regions. Next, a trend analysis was performed to explore the temporal patterns of this disease burden. Linear regression models were used to calculate the estimated annual percentage change (EAPC) values, capturing the changes in the ischemic stroke burden due to lead exposure from 1990 to 2019, both globally and within the various subgroups. To gain a deeper insight into regional disparities, cluster analysis was conducted across 45 GBD regions, grouping them based on similar patterns of disease burden changes. EAPCs for morbidity and mortality were clustered using hierarchical cluster analysis, and countries with similar trends in EAPCs were identified. Finally, to anticipate future patterns in the burden of ischemic stroke linked to lead exposure, the BAPC model was utilized in R with the aid of the BAPC and INLA software packages. This advanced methodology allowed for the concurrent estimation of the effects of age, period, and cohort on the disease burden.

All statistical analyses in this study were performed using R programming (version 4.0.2). A significance level of *P* < 0.05 was applied to all tests, with results below this threshold deemed statistically significant.

## Results

### Global and subtype burden in 2019

In 2019, the number of deaths cases attributed to lead exposure-related ischemic stroke stood at 128,688 (95% CI: 71,550–195,783), accompanied by an ASDR of 1.66 (95% CI: 0.91–2.55). Additionally, the burden of disease expressed in the number of DALYs cases amounted to 2,601,420 (95% CI: 1,470,902–3,919,910), with the age-standardized DALYs rate of 32.21 (95% CI: 18.17–48.71) (Supplementary Tables S1, S2).

**Figure 1:**
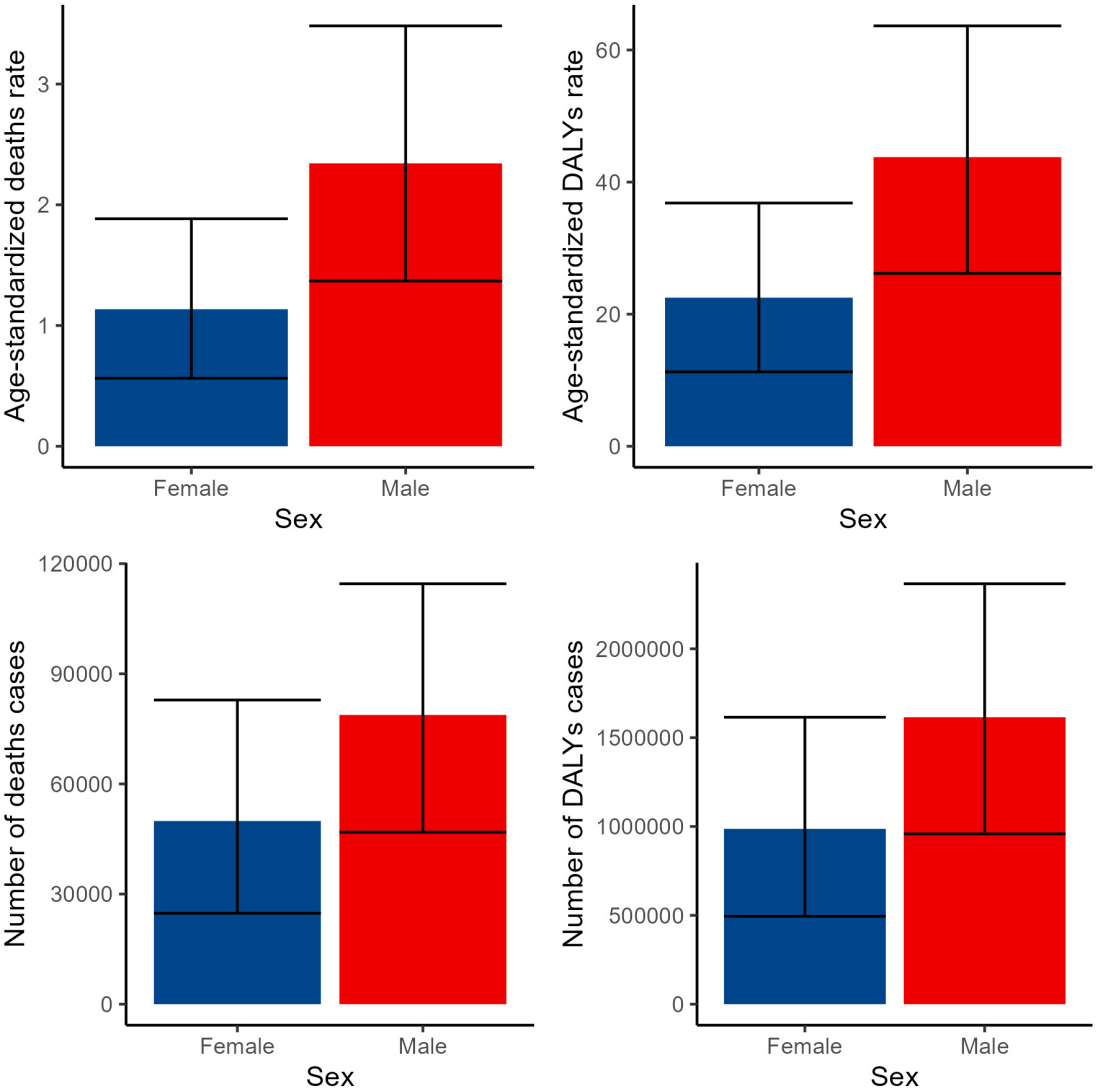
Numbers and ASDR of deaths and DALYs attributable to lead exposure-related ischemic stroke for males and females in 2019. DALYs, disability-adjusted life years.

Our analysis uncovered notable gender disparities in the disease burden, with males experiencing a higher impact across all indicators compared to females. Specifically, the number of deaths among males was 78,790 (95% CI: 46,853–114,521), whereas females had 49,898 deaths (95% CI: 24,764–82,856). ASDR for males was 2.34 (95% CI: 1.37–3.48), significantly higher than the 1.14 (95% CI: 0.56–1.88) observed in females. Similarly, the number of DALYs for males was 1,614,720 (95% CI: 958,481–2,366,017), surpassing the 986,700 (95% CI: 494,641–1,614,923) cases among females. The age-standardized DALY rate for males was 43.78 (95% CI: 26.17–63.65), notably higher than the 22.5 (95% CI: 11.26–36.83) rate for females (Figure 1, Supplementary Tables S1, S2).

**Figure 2:**
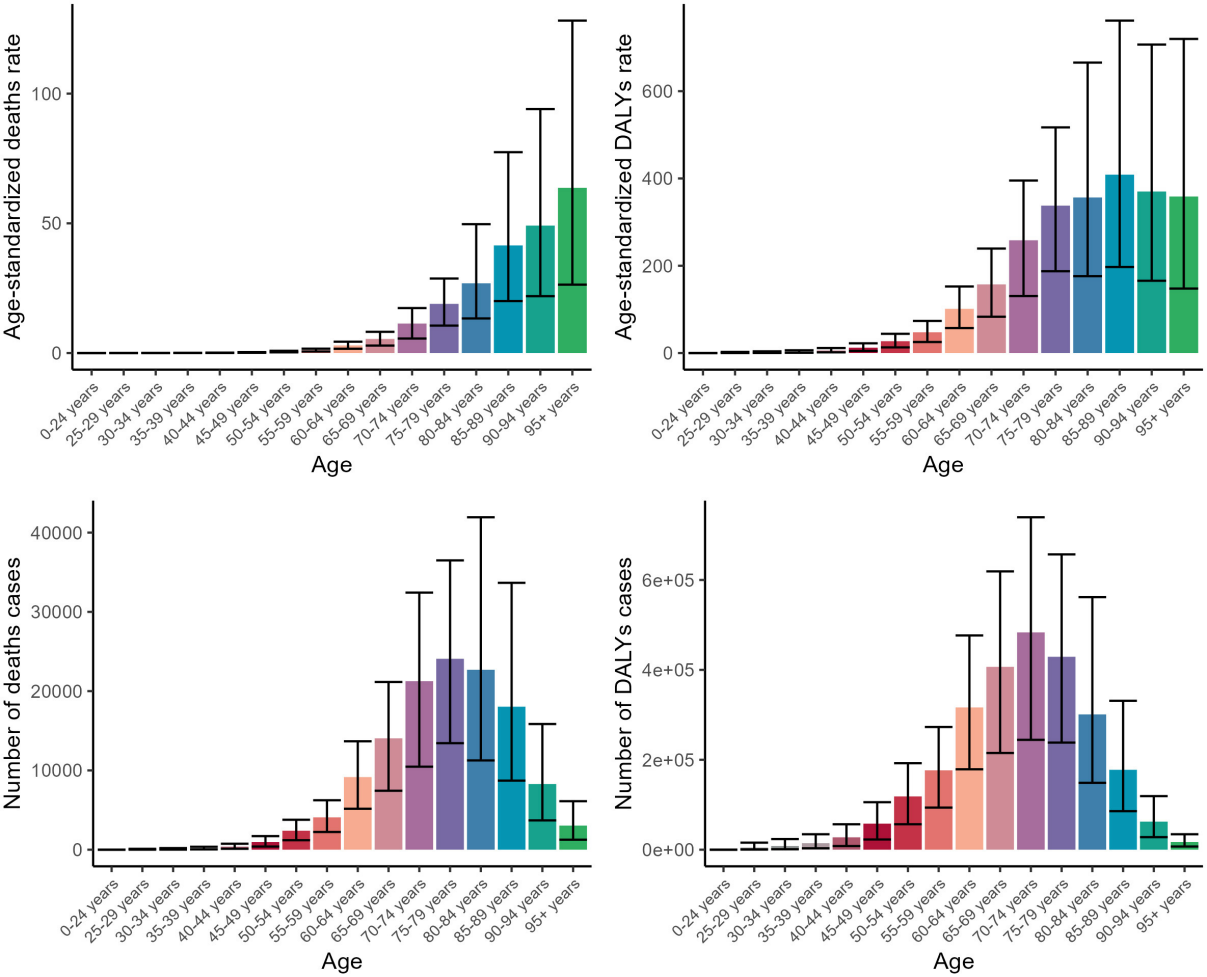
Numbers and ASDR of deaths and DALYs attributable to lead exposure-related ischemic stroke for different age groups in 2019. DALYs, disability-adjusted life years.

In terms of age groups, our analysis of age groups revealed a pronounced elevation in disease burden among middle-aged and older adults. Specifically, the ASDR displayed an upward trend as age progressed, ultimately reaching a peak value of 63.68 (95% CI: 26.36–128.14) among individuals aged 95 and older. In contrast, the three additional indicators exhibited a comparable upward trajectory with age, reaching their peaks in the seventh and eighth decades of life, and subsequently experiencing a minor decline (Figure 2, Supplementary Tables S1, S2).

**Figure 3:**
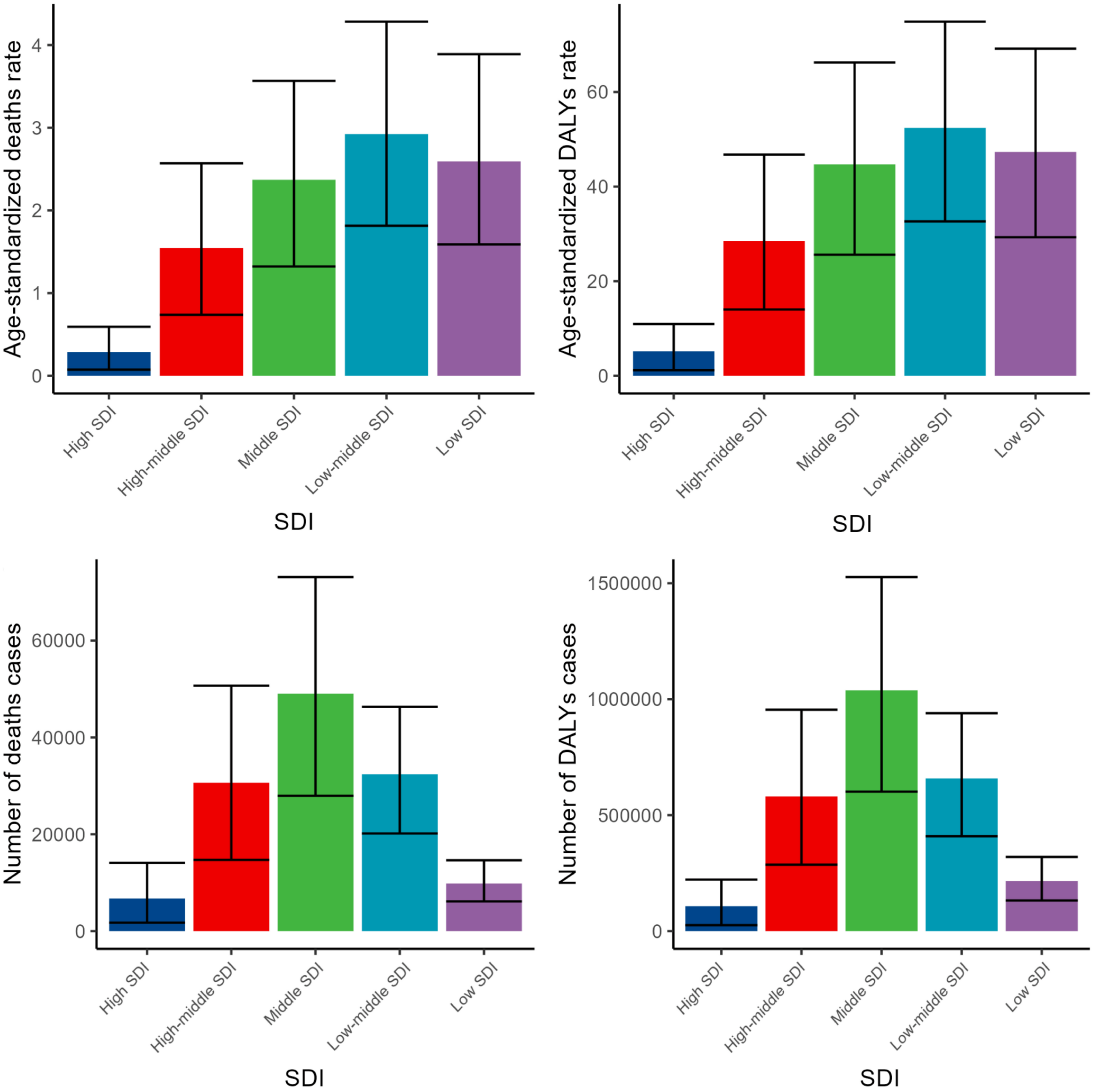
Numbers and ASDR of deaths and DALYs attributable to lead exposure-related ischemic stroke for different SDI regions in 2019. DALYs, disability-adjusted life years.

Across various SDI regions, the disease burden exhibited significant variations, yet each indicator demonstrated a consistent trend. As the level of SDI decreased, the burden of disease correspondingly increased, peaking in the middle SDI region or low-middle SDI region. Subsequently, as the SDI level further declined, the disease burden began to decrease (Figure 3, Supplementary Tables S1, S2).

**Figure 4:**
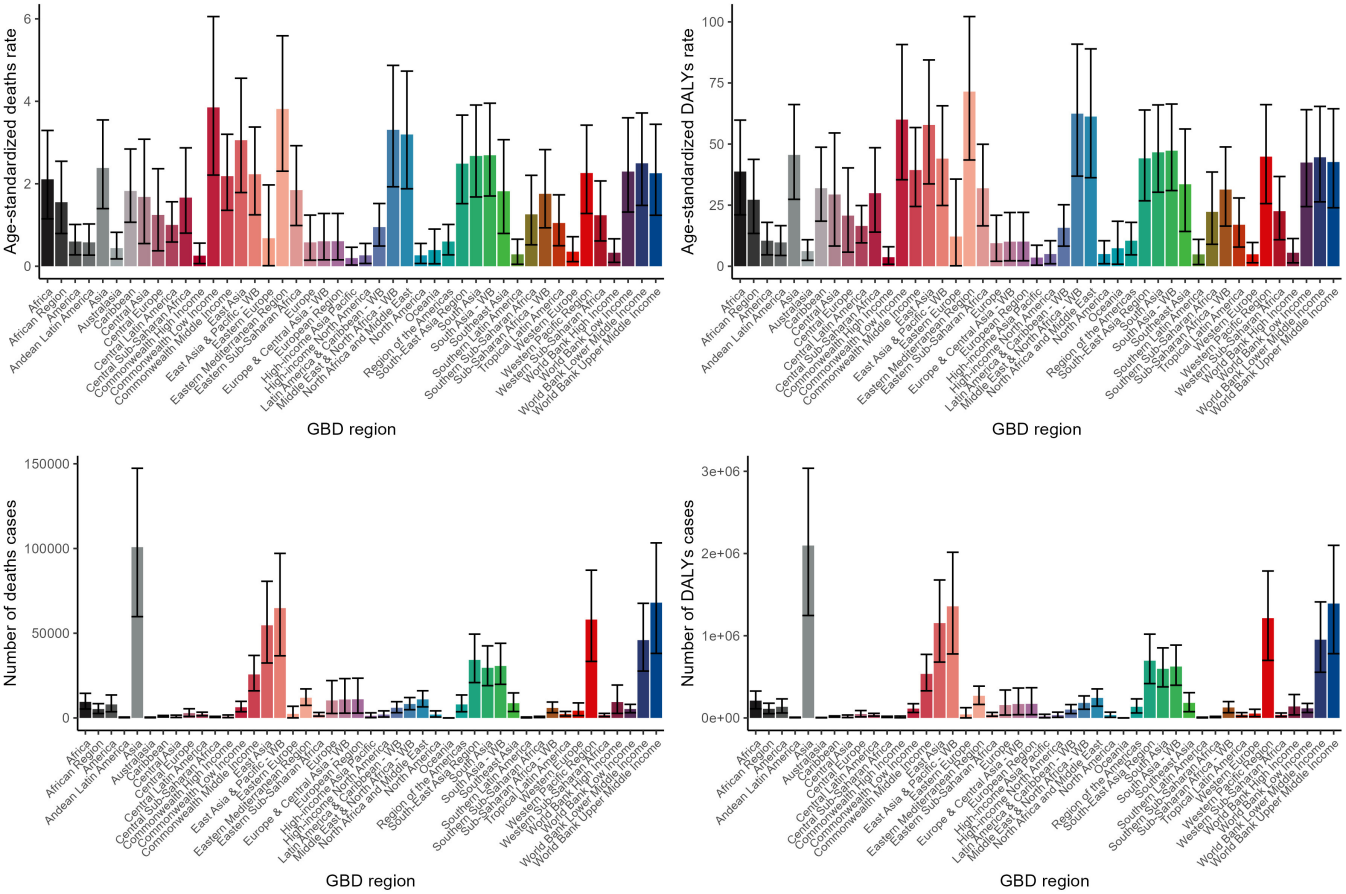
Numbers and ASDR of deaths and DALYs attributable to lead exposure-related ischemic stroke for different GBD regions in 2019. DALYs, disability-adjusted life years.

The burden of lead exposure-related ischemic stroke varied significantly across different GBD regions, with Asia particularly experiencing a markedly higher burden. This was evident in both the number of death cases and disability-adjusted life years (DALYs) (Figure 4, Supplementary Tables S1, S2).

### Temporal trends of the disease burden from 1990 to 2019

Between 1990 and 2019, the age-standardized death and DALY rates for lead exposure-related ischemic stroke initially increased and then subsequently declined. Despite this fluctuation in rates, the absolute number of deaths and DALY cases associated with this condition consistently rose (Supplementary Tables S1, S2).

Analysis of gender-specific trends revealed that the patterns of disease burden closely mirrored the overall trend, with males consistently exhibiting a higher burden of disease compared to females throughout the studied period (Supplementary Figure S1, Supplementary Tables S1, S2).

Examination of different age groups showed that while there were distinct patterns of disease burden variation among them over time, the majority of age cohorts followed the general trend observed (Supplementary Figure S2, Supplementary Tables S1, S2).

From 1990 to 2019, high SDI regions consistently showed a decrease in disease burden across all indicators. In contrast, the other four SDI regions—high-middle, middle, low-middle, and low SDI regions—followed the general trend observed (Supplementary Figure S3, Supplementary Tables S1, S2).

**Figure 5:**
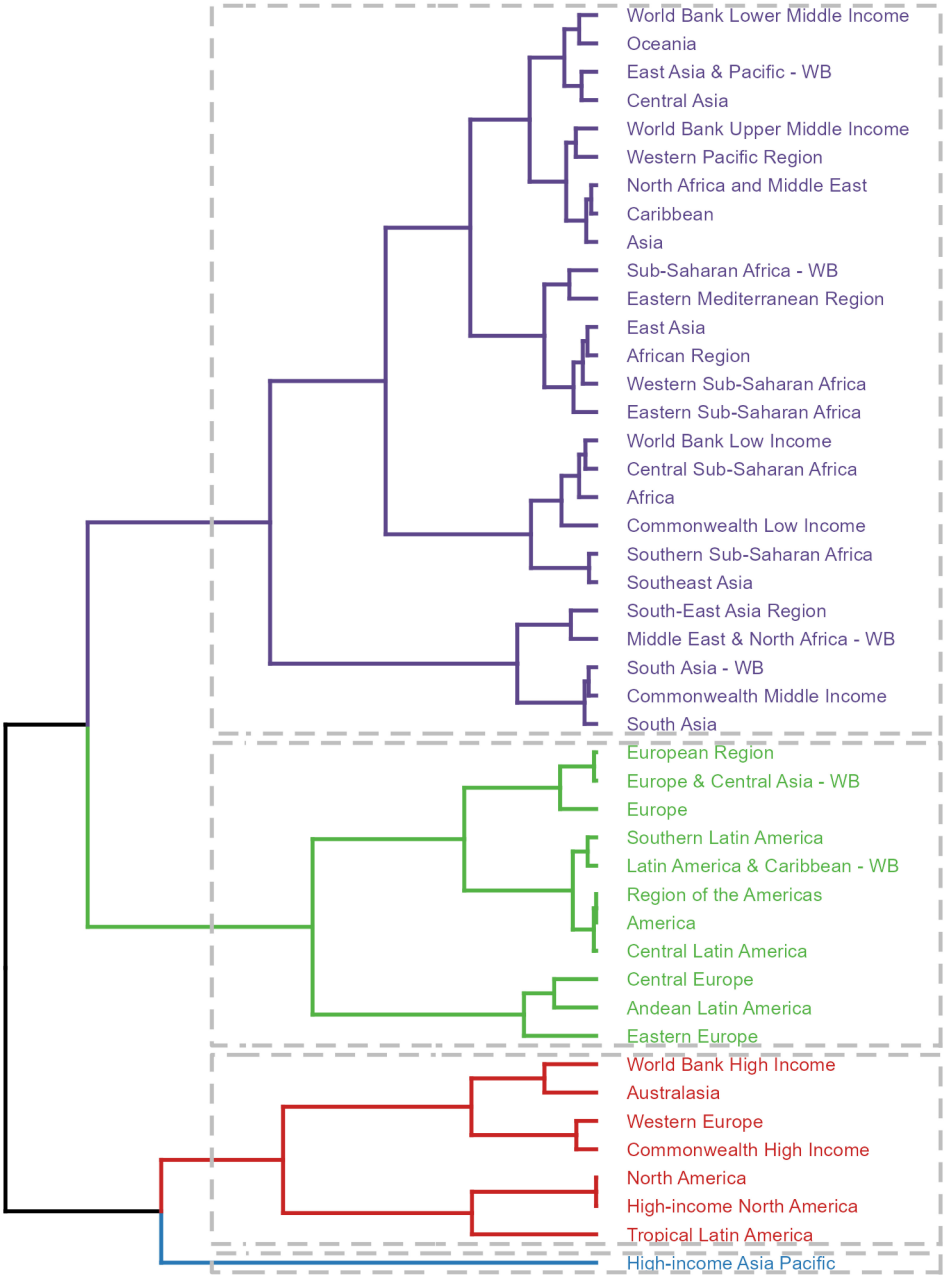
Results of cluster analysis based on the EAPC values of the ASDR for deaths and DALYs attributable to lead exposure-related ischemic stroke from 1990 to 2019. EAPC, estimated annual percentage change; DALYs, disability-adjusted-life-years.

Furthermore, a clustering analysis was conducted on the 45 GBD regions based on the EAPC values derived from the trend models. This analysis identified High-income Asia Pacific as a region belonging to the significant increase group, while six regions, including World Bank Income, fell into the significant decrease group. The specific groupings are presented in the accompanying figure, providing a comprehensive overview of the varying trends in disease burden across different regions (Figure 5, Supplementary Tables S1, S2).

### BAPC analysis predictions

**Figure 6:**
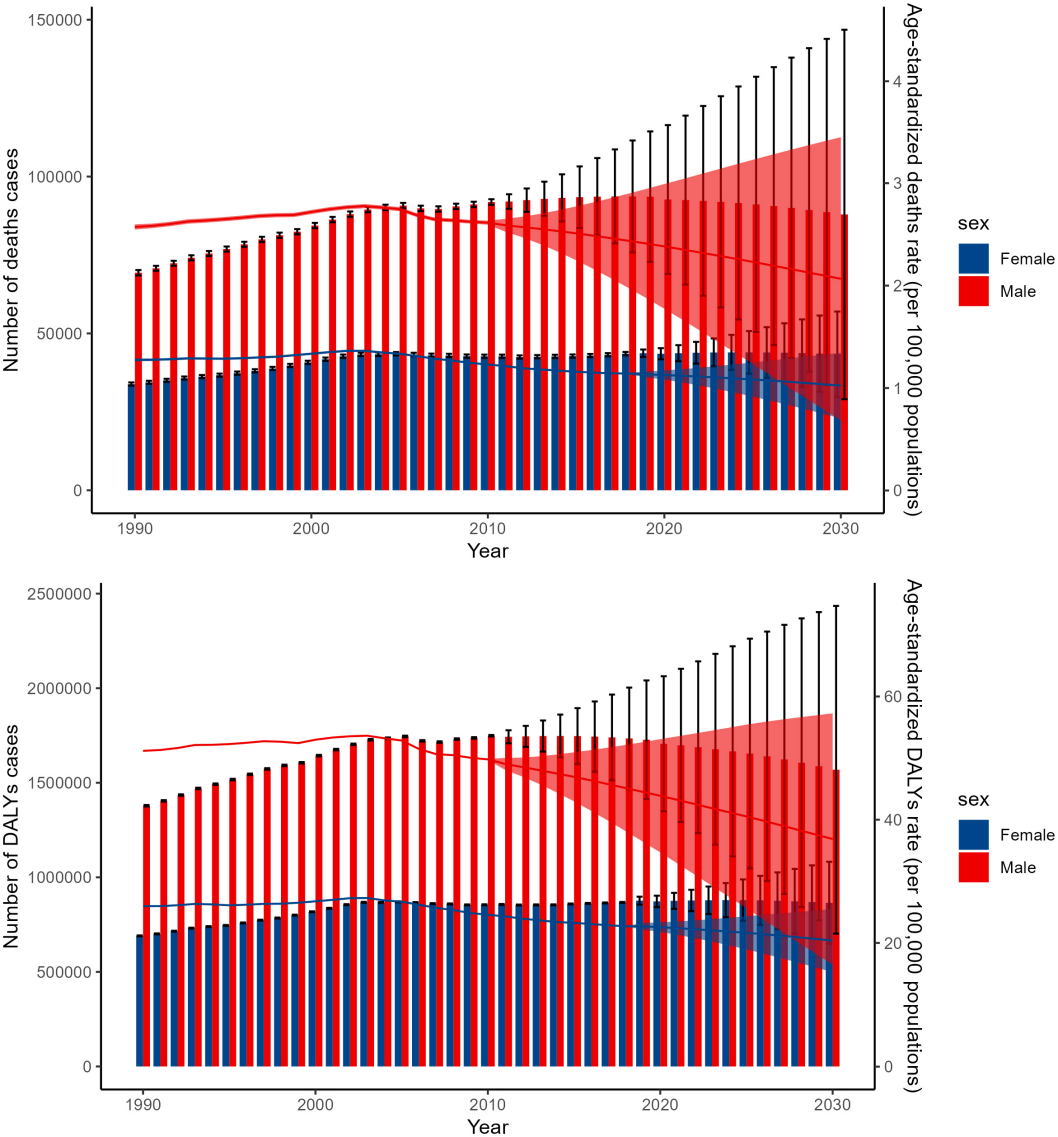
The BAPC model forecasts the global figures and ASDR for deaths and DALYs due to lead exposure-related ischemic stroke from 1990 to 2044. DALYs, disability-adjusted-life-year; BAPC, Bayesian age-period-cohort.

The BAPC model forecasts distinct trends in the global burden of lead exposure-related ischemic stroke for males and females over the next 11 years. For females, the number of deaths and DALYs cases is expected to remain relatively stable, while the age-standardized death and DALY rates are predicted to decline. In contrast, for males, all four indicators—the number of deaths, DALY cases, and the age-standardized death and DALY rates—are projected to decrease (Figure 6).

## Discussion

Cardiovascular diseases account for the highest number of deaths worldwide (33, 34). This study has offered profound insights into the burden associated with lead exposure-related ischemic stroke. Our findings reveal that in 2019, the disease burden attributed to this condition was significant, disproportionately affecting males and middle-aged to older adults. Notably, the heaviest burden was observed in middle and low-middle SDI regions, with Asia particularly affected. The analysis of temporal trends over the past three decades demonstrates a complex pattern of rise and fall in age-standardized death and DALY rates, despite an overall increase in absolute cases. This variation is further triggered by gender-specific differences and region-specific clustering patterns. Our predictions using the BAPC model indicate distinct global trends for males and females, with females anticipated to maintain stable case counts but see decreasing rates, while males are expected to experience declines across all indicators in the next 11 years. These findings emphasized the dire need for targeted interventions to address this preventable condition and underscored the importance of regionally tailored strategies to effectively mitigate this significant health burden (35–37).

Across the investigation, we highlight a substantial health burden from lead exposure-related ischemic stroke in 2019. This condition resulted in 128,688 deaths, with an age-standardized death rate of 1.66. The disease burden was further demonstrated by 2,601,420 DALYs, and an age-standardized DALY rate of 32.21. These findings highlight the urgent need for comprehensive strategies to reduce lead exposure and its related health risks. The broad confidence intervals suggest possible variations in these estimates, stressing the necessity for continuous monitoring and research to enhance our understanding of this critical health issue (38). When examining the temporal trends from 1990 to 2019, we observe an interesting pattern in the age-standardized deaths and DALYs rates. Initially, there was an increase in these rates, suggesting a worsening situation over time. However, subsequent to this initial rise, there was a notable decline, indicating some degree of improvement (39). This fluctuation may reflect changes in exposure levels, improved diagnostic capabilities, or enhanced healthcare interventions aimed at reducing the impact of lead exposure on ischemic stroke. Notably, despite this fluctuation in rates, the absolute number of deaths and DALYs cases associated with lead exposure-related ischemic stroke continued to rise steadily. This finding highlights the need for continued vigilance and efforts to address the underlying causes of this preventable condition (40, 41). It suggests that while progress has been made in reducing the impact of lead exposure on some measures, the overall burden remains significant and requires further action (42).

Our analysis has uncovered profound gender disparities in the burden of disease, with males consistently demonstrating a higher burden across all indicators. The finding that male deaths cases totaled 78,790, significantly outnumbering the 49,898 cases among females, is particularly noteworthy. Similarly, the age-standardized death rate for males, at 2.34, was substantially higher than the rate of 1.14 observed among females. This pattern is further reflected in the number of DALYs cases, where males accounted for 1,614,720 cases, exceeding the 986,700 cases among females. Correspondingly, the age-standardized DALYs rate for males, standing at 43.78, was significantly higher than the rate of 22.5 among females. The analysis of gender-specific trends consistently shows that males bear a higher disease burden compared to females throughout the studied period. This suggests that gender-related factors significantly influence disease outcomes, likely due to a combination of biological, social, and behavioral differences between the sexes (43–45). Potential drivers included biological vulnerability, occupational exposure, or gender-specific healthcare access. These observed gender disparities in disease burden are concerning and warrant further investigation into their underlying causes (46). It is crucial to consider gender-specific risk factors, such as exposure to environmental hazards and disparities in healthcare access and utilization, that may contribute to these differences (47).

Our study found a significant increase in disease burden among middle-aged and older adults, peaking among those aged 95 and above, emphasizing the vulnerability of the older adult to disease-related mortality (48, 49). The burden peaked in the seventh and eighth decades, possibly due to chronic health conditions and decreased resilience (50, 51). Some indicators declined later, potentially reflecting improved healthcare access (52). When analyzing various age groups, it becomes clear that although there are distinct patterns of disease burden variation over time, most age cohorts align with the overall trend. This consistency indicates that common factors contribute to the disease burden across all ages (53, 54). However, it is crucial to acknowledge that each age group faces unique challenges and needs, which require tailored healthcare strategies to address their specific requirements. Developing age-specific approaches is essential to effectively manage and mitigate the disease burden within each demographic.

The analysis of disease burden across various SDI regions showed significant variations in disease burden, peaking in middle and low-middle SDI regions. This trend highlights the impact of social, economic, and infrastructural disparities on disease burden (55). Limited healthcare access, ongoing industrialization, and high exposure to or informal recycling activities contribute to this burden (56). Regional disparities in disease measurement and surveillance methods might contribute to inconsistent data collection, potentially distorting the true vascular diseases burden in low SDI regions (57). Examining trends in high SDI regions reveals a consistent decrease in disease burden across all indicators from 1990 to 2019. This trend suggests that improvements in healthcare, environmental regulations, and economic prosperity have significantly reduced the disease burden in these areas. Factors such as enhanced healthcare access, better living conditions, and increased disease awareness and prevention measures likely contribute to this decline (58). This uniformity indicates that common factors influence disease burden across various SDI levels. However, it's crucial to recognize that each region faces distinct challenges and opportunities, underscoring the need for tailored strategies to meet their specific healthcare needs (59).

The notable regional variations in the disease burden associated with lead exposure-related ischemic stroke across different GBD regions are intriguing and deserve further discussion. Asia, in particular, stands out as a region with a significantly higher burden, both in terms of the number of deaths and DALYs cases, compared to other regions. This finding is concerning and suggests that lead exposure may be a particularly significant public health issue in Asia, which has been reported before (60). The clustering analysis of the 45 GBD regions, utilizing EAPC values derived from trend models, reveals diverse trends in disease burden. Notably, High-income Asia Pacific is identified as a region with a significant increase in lead exposure-related ischemic stroke, despite its relatively high income and advanced healthcare resources. This indicates that specific challenges or vulnerabilities within this sub-region need to be addressed. Conversely, six regions, including those classified by World Bank Income, show a significant decrease in disease burden. This suggests successful implementation of measures to mitigate the disease burden in these areas. However, even in these regions, the burden remains significant, highlighting the need for continued efforts to achieve substantial reductions.

The BAPC model's projections for the global burden of lead exposure-related ischemic stroke over the next 11 years reveal noteworthy trends among males and females. For females, the model predicts stable death and DALYs cases, suggesting limited progress in reducing the burden despite efforts to reduce lead exposure. However, the ASDR are predicted to decline, indicating a relative decrease in burden. In contrast, males are predicted to experience a decline in all indicators, indicating significant progress in reducing both the absolute and relative burden. These gender-specific differences may reflect exposure patterns or healthcare utilization (61). Males ‘higher burden could be due to environmental or occupational exposures, while females' more stable burden may reflect better healthcare access (61). These findings highlight the need for targeted interventions to address gender-specific vulnerabilities.

Future research could build upon our findings by conducting longitudinal studies to monitor changes in lead exposure and its health impacts over time. Investigating the biological mechanisms underlying lead-related ischemic stroke could provide deeper insights into the pathophysiology of the disease. Additionally, expanding research to include diverse populations and multiple centers would enhance the generalizability of the results. Through integrating individual-level data on blood lead levels, deep exploration of the interaction between lead and other cardiovascular risk factors was conducted. Further exploration into effective interventions and policies to reduce lead exposure and mitigate its health effects is crucial (62). These efforts could significantly advance our understanding and management of lead-related health risks. Although our study provides valuable insights into the burden of lead-related ischemic stroke, it is not without limitations. One major constraint is the complex nature of lead exposure and its interaction with other risk factors for ischemic stroke poses challenges in accurately assessing the overall burden. There exist potential biases in GBD data and the uncertainty in lead exposure attribution. The use of BAPC analysis, while powerful, also has its limitations in capturing the full range of potential confounders and interactions (63). For example, the model assumptions in BAPC could introduce prediction errors due to absent population-level biomonitoring of BLL. Further sensitivity analysis was needed to assess the robustness of findings to different model assumptions or data sources. Furthermore, our predictions are based on current trends and assumptions, which may not fully account for future changes in exposure patterns, population demographics, or healthcare systems (64, 65).

## Conclusion

In conclusion, the findings of this study highlight the significant burden of lead exposure-related ischemic stroke globally and the need for urgent and targeted interventions. Future research should focus on exploring the underlying mechanisms of lead-related stroke, evaluating the effectiveness of prevention and treatment strategies, and enhancing the capacity of healthcare systems to address this growing burden.

## Data Availability

The original contributions presented in the study are included in the article/Supplementary material, further inquiries can be directed to the corresponding author/s.
