# Supplementary material for: The global burden of lead exposure-related ischemic stroke: based on Bayesian age-period-cohort analysis
Source: Front Public Health. 2025 Jul 16;13:1608129. doi: 10.3389/fpubh.2025.1608129 (PMC12307299; doi:10.3389/fpubh.2025.1608129)
Supplement: Supplementary file 1 [file Table_1.docx]

| Table S1:The number of deaths cases and the age-standardized deaths rate of lead exposure-related ischemic stroke in 1990 and 2019, and its trends from 1990 to 2019 globally | | | | | |  |
| --- | --- | --- | --- | --- | --- | --- |
|  | Num_1990 | ASR_1990 | Num_2019 | ASR_2019 | EAPC_CI |  |
| Global | 62107 (32309-99550) | 1.81 (0.92-2.98) | 128688 (71550-195783) | 1.66 (0.91-2.55) | -0.36 (-0.51--0.2) |  |
| Sex |  |  |  |  |  |  |
| Female | 24636 (11014-44018) | 1.27 (0.55-2.3) | 49898 (24764-82856) | 1.14 (0.56-1.88) | -0.48 (-0.65--0.32) |  |
| Male | 37470 (21056-57166) | 2.56 (1.41-3.99) | 78790 (46853-114521) | 2.34 (1.37-3.48) | -0.34 (-0.5--0.19) |  |
| Age |  |  |  |  |  |  |
| 0-24 years | 0 (0-0) | 0.00 (0.00-0.00) | 0 (0-0) | 0.00 (0.00-0.00) | 0.00 (0.00-0.00) |  |
| 25-29 years | 77 (19-178) | 0.02 (0-0.04) | 32 (2-108) | 0.01 (0-0.02) | -4.32 (-4.51--4.12) |  |
| 30-34 years | 134 (46-269) | 0.03 (0.01-0.07) | 73 (9-201) | 0.01 (0-0.03) | -3.84 (-4.16--3.51) |  |
| 35-39 years | 255 (106-464) | 0.07 (0.03-0.13) | 159 (36-366) | 0.03 (0.01-0.07) | -3.06 (-3.43--2.69) |  |
| 40-44 years | 471 (219-800) | 0.16 (0.08-0.28) | 376 (115-750) | 0.08 (0.02-0.15) | -2.52 (-2.84--2.21) |  |
| 45-49 years | 896 (454-1466) | 0.39 (0.2-0.63) | 967 (396-1712) | 0.2 (0.08-0.36) | -2.02 (-2.27--1.76) |  |
| 50-54 years | 1936 (1050-3073) | 0.91 (0.49-1.45) | 2392 (1196-3776) | 0.55 (0.27-0.86) | -1.77 (-1.94--1.6) |  |
| 55-59 years | 3214 (1835-4959) | 1.73 (0.99-2.67) | 4080 (2233-6236) | 1.1 (0.6-1.68) | -1.51 (-1.65--1.38) |  |
| 60-64 years | 6673 (3691-10101) | 4.15 (2.3-6.29) | 9160 (5163-13676) | 2.93 (1.65-4.38) | -1.24 (-1.36--1.12) |  |
| 65-69 years | 8332 (4200-13221) | 6.75 (3.4-10.71) | 14051 (7434-21155) | 5.43 (2.88-8.18) | -0.87 (-1.1--0.65) |  |
| 70-74 years | 11132 (5102-18216) | 13.17 (6.04-21.55) | 21261 (10470-32430) | 11.36 (5.6-17.33) | -0.55 (-0.79--0.31) |  |
| 75-79 years | 11898 (5631-19865) | 19.41 (9.18-32.4) | 24082 (13437-36495) | 18.95 (10.58-28.72) | -0.11 (-0.31-0.09) |  |
| 80-84 years | 8610 (3410-17445) | 24.45 (9.68-49.53) | 22695 (11261-41936) | 26.88 (13.34-49.67) | 0.36 (0.21-0.51) |  |
| 85-89 years | 5497 (2051-11385) | 36.48 (13.61-75.56) | 18038 (8711-33665) | 41.48 (20.03-77.42) | 0.38 (0.24-0.51) |  |
| 90-94 years | 2231 (798-4722) | 50.63 (18.11-107.18) | 8282 (3696-15854) | 49.13 (21.92-94.05) | -0.27 (-0.37--0.17) |  |
| 95+ years | 752 (270-1600) | 73.06 (26.26-155.42) | 3040 (1258-6116) | 63.68 (26.36-128.14) | -0.62 (-0.7--0.54) |  |
| SDI region |  |  |  |  |  |  |
| High SDI | 8433 (2494-16441) | 0.81 (0.24-1.57) | 6722 (1745-14101) | 0.29 (0.07-0.59) | -3.98 (-4.16--3.8) |  |
| High-middle SDI | 16930 (7497-30691) | 1.82 (0.77-3.36) | 30637 (14712-50684) | 1.55 (0.74-2.57) | -0.69 (-0.93--0.44) |  |
| Middle SDI | 19980 (11730-30126) | 2.47 (1.4-3.74) | 49033 (27945-73121) | 2.37 (1.32-3.57) | -0.09 (-0.29-0.1) |  |
| Low-middle SDI | 12668 (7474-18982) | 2.84 (1.68-4.28) | 32395 (20168-46322) | 2.92 (1.81-4.28) | 0.02 (-0.19-0.23) |  |
| Low SDI | 4066 (2379-6347) | 2.38 (1.35-3.87) | 9840 (6139-14641) | 2.59 (1.59-3.89) | 0.27 (0.11-0.44) |  |
| GBD region |  |  |  |  |  |  |
| Africa | 4064 (2228-6324) | 1.88 (1.01-3) | 9493 (5291-14529) | 2.11 (1.15-3.29) | 0.5 (0.29-0.7) |  |
| African Region | 2411 (1170-3981) | 1.45 (0.69-2.41) | 5256 (2636-8437) | 1.55 (0.79-2.55) | 0.23 (0.05-0.41) |  |
| America | 6279 (2931-10382) | 1.07 (0.5-1.76) | 7982 (3668-13554) | 0.6 (0.28-1.01) | -2.14 (-2.2--2.07) |  |
| Andean Latin America | 149 (67-247) | 0.86 (0.39-1.44) | 304 (139-535) | 0.58 (0.27-1.03) | -1.42 (-1.57--1.28) |  |
| Asia | 40520 (23788-61221) | 2.53 (1.43-3.93) | 100809 (59791-147360) | 2.38 (1.4-3.55) | -0.22 (-0.42--0.02) |  |
| Australasia | 256 (111-437) | 1.17 (0.51-2.05) | 265 (109-499) | 0.44 (0.18-0.82) | -3.88 (-4.07--3.69) |  |
| Caribbean | 477 (272-718) | 2 (1.12-3.03) | 947 (553-1474) | 1.83 (1.07-2.84) | -0.16 (-0.23--0.1) |  |
| Central Asia | 607 (155-1133) | 1.49 (0.39-2.78) | 887 (271-1643) | 1.68 (0.55-3.08) | 0.07 (-0.4-0.55) |  |
| Central Europe | 2349 (530-4673) | 1.76 (0.39-3.52) | 2853 (864-5423) | 1.24 (0.37-2.37) | -1.56 (-1.9--1.23) |  |
| Central Latin America | 1171 (713-1697) | 1.74 (1.04-2.59) | 2193 (1283-3398) | 1 (0.59-1.56) | -2.14 (-2.22--2.05) |  |
| Central Sub-Saharan Africa | 222 (94-386) | 1.46 (0.61-2.56) | 568 (267-940) | 1.66 (0.8-2.87) | 0.45 (0.35-0.55) |  |
| Commonwealth High Income | 1158 (272-2347) | 0.76 (0.18-1.55) | 795 (197-1749) | 0.26 (0.06-0.56) | -4.29 (-4.5--4.07) |  |
| Commonwealth Low Income | 2280 (1337-3454) | 3.49 (2.05-5.34) | 6329 (3659-9787) | 3.85 (2.21-6.06) | 0.61 (0.13-1.09) |  |
| Commonwealth Middle Income | 10787 (6378-16644) | 2.42 (1.4-3.77) | 25684 (16005-36887) | 2.18 (1.36-3.2) | -0.65 (-0.91--0.4) |  |
| East Asia | 20860 (12491-31025) | 2.97 (1.72-4.48) | 54675 (32428-80652) | 3.06 (1.79-4.56) | 0.24 (-0.01-0.49) |  |
| East Asia & Pacific - WB | 25543 (14401-38630) | 2.3 (1.24-3.63) | 64824 (36680-97120) | 2.23 (1.25-3.38) | 0 (-0.2-0.19) |  |
| Eastern Europe | 2176 (2-6850) | 0.86 (0-2.69) | 2384 (46-6885) | 0.68 (0.01-1.97) | -1.53 (-2.11--0.95) |  |
| Eastern Mediterranean Region | 5280 (3183-7887) | 3.6 (2.15-5.37) | 11931 (7417-17134) | 3.81 (2.31-5.59) | 0.18 (-0.06-0.43) |  |
| Eastern Sub-Saharan Africa | 942 (503-1509) | 1.79 (0.95-2.87) | 2028 (1063-3225) | 1.85 (0.99-2.92) | 0.14 (0-0.28) |  |
| Europe | 11200 (2835-23832) | 1.12 (0.28-2.39) | 10326 (2649-22053) | 0.58 (0.15-1.24) | -2.65 (-2.88--2.41) |  |
| Europe & Central Asia - WB | 11614 (2956-24536) | 1.13 (0.29-2.41) | 10897 (2821-23221) | 0.61 (0.15-1.28) | -2.54 (-2.79--2.29) |  |
| European Region | 11697 (2978-24689) | 1.13 (0.29-2.41) | 11030 (2857-23461) | 0.61 (0.16-1.28) | -2.53 (-2.78--2.28) |  |
| High-income Asia Pacific | 1422 (262-3003) | 0.82 (0.14-1.75) | 1274 (189-3031) | 0.2 (0.03-0.46) | -5.37 (-5.54--5.2) |  |
| High-income North America | 2508 (944-4554) | 0.67 (0.25-1.21) | 1988 (524-4179) | 0.26 (0.07-0.55) | -3.61 (-3.82--3.4) |  |
| Latin America & Caribbean - WB | 3785 (1992-5926) | 1.69 (0.88-2.69) | 6010 (3104-9601) | 0.95 (0.49-1.52) | -2.05 (-2.09--2.01) |  |
| Middle East & North Africa - WB | 3580 (2232-5191) | 3.68 (2.19-5.46) | 8229 (4866-12041) | 3.31 (1.93-4.87) | -0.34 (-0.53--0.15) |  |
| North Africa and Middle East | 4729 (2842-6911) | 3.46 (2.05-5.16) | 10947 (6544-16045) | 3.2 (1.88-4.73) | -0.19 (-0.39-0.01) |  |
| North America | 2508 (944-4554) | 0.67 (0.25-1.21) | 1988 (524-4179) | 0.26 (0.07-0.55) | -3.61 (-3.82--3.4) |  |
| Oceania | 7 (1-18) | 0.39 (0.05-0.94) | 17 (2-41) | 0.39 (0.06-0.9) | -0.06 (-0.15-0.02) |  |
| Region of the Americas | 6279 (2931-10382) | 1.07 (0.5-1.76) | 7982 (3668-13554) | 0.6 (0.28-1.01) | -2.14 (-2.2--2.07) |  |
| South-East Asia Region | 13188 (7670-20085) | 2.73 (1.58-4.19) | 34256 (20877-49475) | 2.49 (1.52-3.67) | -0.47 (-0.73--0.22) |  |
| South Asia | 11842 (7087-18120) | 3.05 (1.83-4.74) | 29591 (19068-42535) | 2.68 (1.68-3.91) | -0.71 (-1.02--0.41) |  |
| South Asia - WB | 12306 (7336-18917) | 3.06 (1.83-4.78) | 30682 (19838-44056) | 2.69 (1.7-3.95) | -0.68 (-0.98--0.37) |  |
| Southeast Asia | 3061 (1288-5225) | 1.58 (0.64-2.73) | 8705 (3789-14774) | 1.82 (0.8-3.07) | 0.71 (0.49-0.92) |  |
| Southern Latin America | 209 (10-493) | 0.49 (0.02-1.17) | 251 (42-571) | 0.29 (0.05-0.66) | -2.01 (-2.13--1.89) |  |
| Southern Sub-Saharan Africa | 235 (96-406) | 1.05 (0.42-1.85) | 544 (222-949) | 1.26 (0.52-2.2) | 0.74 (0.24-1.25) |  |
| Sub-Saharan Africa - WB | 2694 (1378-4354) | 1.61 (0.81-2.62) | 5901 (3104-9388) | 1.76 (0.93-2.83) | 0.31 (0.1-0.52) |  |
| Tropical Latin America | 1794 (898-2832) | 2.48 (1.22-3.97) | 2348 (1110-3824) | 1.05 (0.5-1.73) | -2.93 (-2.98--2.88) |  |
| Western Europe | 6308 (2132-11861) | 1.05 (0.35-1.99) | 4294 (1371-8867) | 0.35 (0.11-0.72) | -4.2 (-4.37--4.02) |  |
| Western Pacific Region | 23158 (13191-35679) | 2.38 (1.29-3.73) | 58068 (33362-87207) | 2.26 (1.28-3.42) | -0.08 (-0.28-0.11) |  |
| Western Sub-Saharan Africa | 783 (331-1385) | 1.17 (0.49-2.08) | 1625 (802-2693) | 1.24 (0.61-2.07) | 0.17 (0.01-0.33) |  |
| World Bank High Income | 11983 (3808-22760) | 0.93 (0.3-1.77) | 9368 (2698-19420) | 0.33 (0.09-0.67) | -4.02 (-4.19--3.85) |  |
| World Bank Low Income | 2246 (1231-3531) | 2.07 (1.11-3.29) | 5266 (3069-8014) | 2.3 (1.31-3.6) | 0.42 (0.26-0.58) |  |
| World Bank Lower Middle Income | 18514 (10294-29072) | 2.39 (1.31-3.78) | 45940 (27655-67648) | 2.5 (1.48-3.72) | 0.03 (-0.22-0.27) |  |
| World Bank Upper Middle Income | 29335 (16050-46387) | 2.33 (1.22-3.81) | 68053 (38093-103310) | 2.26 (1.24-3.44) | -0.08 (-0.3-0.15) |  |

| Table S2：The number of DALYs cases and the age-standardized DALYs rate of lead exposure-related ischemic stroke in 1990 and 2019, and its trends from 1990 to 2019 globally. Abbreviations: DALYs, disability-adjusted-life-years. | | | | | |
| --- | --- | --- | --- | --- | --- |
|  | Num_1990 | ASR_1990 | Num_2019 | ASR_2019 | EAPC_CI |
| Global | 1425183 (762067-2199251) | 37.21 (19.87-58.27) | 2601420 (1470902-3919910) | 32.21 (18.17-48.71) | -0.53 (-0.68--0.39) |
| Sex |  |  |  |  |  |
| Female | 541654 (248114-902842) | 25.98 (11.89-43.41) | 986700 (494641-1614923) | 22.5 (11.26-36.83) | -0.56 (-0.71--0.42) |
| Male | 883529 (504156-1316747) | 51.12 (29.34-76.66) | 1614720 (958481-2366017) | 43.78 (26.17-63.65) | -0.55 (-0.7--0.4) |
| Age |  |  |  |  |  |
| 0-24 years | 10387 (2528-24522) | 2.35 (0.57-5.54) | 4226 (232-15623) | 0.7 (0.04-2.58) | -4.36 (-4.49--4.24) |
| 25-29 years | 14656 (4925-30440) | 3.8 (1.28-7.89) | 8150 (902-23654) | 1.35 (0.15-3.93) | -3.77 (-4.04--3.49) |
| 30-34 years | 22741 (9400-41833) | 6.45 (2.66-11.86) | 14523 (3107-34341) | 2.68 (0.57-6.35) | -2.99 (-3.3--2.67) |
| 35-39 years | 32652 (14709-56768) | 11.41 (5.14-19.83) | 27518 (8111-56549) | 5.58 (1.64-11.46) | -2.36 (-2.63--2.08) |
| 40-44 years | 50460 (25474-82632) | 21.71 (10.96-35.55) | 57951 (22691-105639) | 12.23 (4.79-22.3) | -1.82 (-2.04--1.6) |
| 45-49 years | 89719 (48719-140242) | 42.2 (22.92-65.97) | 118518 (56598-192436) | 27.13 (12.96-44.05) | -1.54 (-1.69--1.39) |
| 50-54 years | 129561 (74291-198228) | 69.88 (40.07-106.91) | 176571 (93639-272958) | 47.59 (25.24-73.57) | -1.27 (-1.4--1.15) |
| 55-59 years | 217599 (120697-328222) | 135.45 (75.13-204.32) | 316485 (178856-476614) | 101.26 (57.23-152.5) | -1.02 (-1.14--0.91) |
| 60-64 years | 227770 (113970-358972) | 184.45 (92.29-290.69) | 406775 (215102-619110) | 157.31 (83.18-239.42) | -0.67 (-0.86--0.47) |
| 65-69 years | 241756 (110932-392240) | 286.06 (131.26-464.12) | 483402 (244355-739505) | 258.38 (130.61-395.27) | -0.39 (-0.6--0.18) |
| 70-74 years | 203030 (95072-340742) | 331.16 (155.07-555.78) | 429131 (238242-656920) | 337.76 (187.51-517.04) | 0.04 (-0.14-0.22) |
| 75-79 years | 110881 (44103-224241) | 314.83 (125.22-636.69) | 300941 (148688-561841) | 356.47 (176.12-665.51) | 0.47 (0.33-0.61) |
| 80-84 years | 53172 (19900-109895) | 352.87 (132.06-729.29) | 177718 (85718-331198) | 408.73 (197.14-761.71) | 0.46 (0.33-0.58) |
| 85-89 years | 16564 (5922-34817) | 375.93 (134.41-790.22) | 62391 (27917-119152) | 370.1 (165.6-706.81) | -0.19 (-0.28--0.1) |
| 90-94 years | 4234 (1511-8968) | 411.31 (146.8-871.19) | 17120 (7051-34354) | 358.67 (147.72-719.72) | -0.6 (-0.68--0.53) |
| 95+ years |  |  |  |  |  |
| SDI region | 150968 (44421-293727) | 14.21 (4.18-27.57) | 107403 (25628-222276) | 5.18 (1.18-10.94) | -3.77 (-3.91--3.63) |
| High SDI | 374195 (172084-646636) | 36.18 (16.58-62.74) | 580556 (286510-954472) | 28.5 (14.01-46.77) | -0.95 (-1.18--0.73) |
| High-middle SDI | 498627 (293445-726727) | 51.49 (30.2-76.53) | 1038162 (601288-1526743) | 44.71 (25.59-66.25) | -0.44 (-0.59--0.28) |
| Middle SDI | 301363 (181954-442064) | 55.24 (33.61-81.59) | 658321 (409049-939612) | 52.41 (32.65-74.88) | -0.22 (-0.4--0.05) |
| Low-middle SDI | 99414 (58138-153177) | 46.65 (27.24-70.93) | 215832 (132132-319862) | 47.34 (29.29-69.17) | 0.03 (-0.12-0.19) |
| Low SDI |  |  |  |  |  |
| GBD region | 99558 (53103-153071) | 37.94 (20.67-58.3) | 210496 (111270-327060) | 38.75 (21.03-59.8) | 0.19 (-0.01-0.39) |
| Africa | 58097 (27319-94965) | 28.73 (13.83-46.65) | 111028 (52353-179094) | 27.21 (13.42-43.73) | -0.19 (-0.37--0.02) |
| African Region | 126752 (58275-204266) | 20.92 (9.71-33.68) | 135763 (60835-231778) | 10.45 (4.66-17.95) | -2.49 (-2.54--2.44) |
| America | 3103 (1330-5139) | 16 (7.02-26.24) | 5281 (2357-8989) | 9.76 (4.41-16.61) | -1.82 (-1.97--1.67) |
| Andean Latin America | 999443 (592473-1456956) | 52.27 (30.94-76.84) | 2097093 (1246060-3037074) | 45.56 (27.38-66.17) | -0.48 (-0.65--0.31) |
| Asia | 4356 (1965-7195) | 18.7 (8.32-31.14) | 3424 (1362-6037) | 6.18 (2.41-10.85) | -4.2 (-4.35--4.05) |
| Australasia | 9743 (5591-14480) | 38.09 (21.83-56.36) | 16498 (9556-25127) | 31.93 (18.45-48.71) | -0.47 (-0.53--0.42) |
| Caribbean | 13071 (3111-24794) | 29.36 (7.1-55.26) | 18497 (4827-35679) | 29.37 (8.27-54.54) | -0.34 (-0.81-0.13) |
| Central Asia | 48008 (11137-92885) | 33.22 (7.63-64.71) | 46821 (13344-89931) | 20.72 (5.77-40.25) | -1.99 (-2.32--1.65) |
| Central Europe | 24495 (15047-34541) | 31.78 (19.84-44.72) | 37176 (21751-55872) | 16.5 (9.57-24.84) | -2.48 (-2.56--2.4) |
| Central Latin America | 5692 (2354-9839) | 28.83 (12.09-49.21) | 12692 (5763-20682) | 29.9 (13.97-48.5) | 0.12 (0.02-0.21) |
| Central Sub-Saharan Africa | 19105 (4564-38800) | 12.18 (2.88-24.6) | 10631 (2455-22673) | 3.7 (0.82-7.97) | -4.56 (-4.74--4.38) |
| Commonwealth High Income | 48696 (29016-73188) | 62.45 (37.44-92.13) | 113629 (66538-173008) | 60.01 (35.4-90.73) | 0.18 (-0.22-0.57) |
| Commonwealth Low Income | 259457 (154465-395062) | 46.91 (27.95-70.83) | 535687 (329923-773054) | 39.36 (24.45-56.76) | -0.83 (-1.04--0.62) |
| Commonwealth Middle Income | 536044 (323824-770775) | 63.73 (39-93.28) | 1153806 (679280-1678276) | 57.83 (33.7-84.42) | -0.24 (-0.44--0.04) |
| East Asia | 642315 (368653-949743) | 49.46 (28.29-74.63) | 1357694 (778456-2015410) | 44.04 (24.86-65.66) | -0.32 (-0.48--0.16) |
| East Asia & Pacific - WB | 43716 (41-136178) | 15.89 (0.02-49.58) | 42721 (671-124834) | 12.16 (0.19-35.67) | -1.61 (-2.23--0.99) |
| Eastern Europe | 128926 (80747-188494) | 74.35 (46.18-108.15) | 269612 (164060-386923) | 71.47 (43.48-102.18) | -0.13 (-0.38-0.12) |
| Eastern Mediterranean Region | 22509 (11863-36236) | 34.61 (18.72-54.7) | 41989 (20905-67111) | 31.91 (16.53-49.92) | -0.27 (-0.41--0.14) |
| Eastern Sub-Saharan Africa | 198585 (47993-429162) | 19.03 (4.62-40.99) | 156697 (35495-339702) | 9.43 (2.03-20.86) | -2.78 (-3.06--2.5) |
| Europe | 207801 (50285-445074) | 19.41 (4.69-41.55) | 169649 (39386-363757) | 10.06 (2.23-21.99) | -2.64 (-2.92--2.35) |
| Europe & Central Asia - WB | 209332 (50701-448082) | 19.42 (4.7-41.56) | 171760 (39908-367864) | 10.09 (2.24-22.04) | -2.63 (-2.92--2.34) |
| European Region | 27425 (5179-56208) | 14.31 (2.68-29.74) | 19012 (2496-44669) | 3.55 (0.42-8.6) | -5.2 (-5.36--5.04) |
| High-income Asia Pacific | 47232 (16907-84365) | 12.84 (4.5-22.93) | 34086 (7297-70354) | 4.99 (1.02-10.44) | -3.42 (-3.54--3.3) |
| High-income North America | 79751 (40916-121862) | 31.6 (16.46-48.54) | 101902 (53349-162484) | 15.74 (8.22-25.17) | -2.48 (-2.53--2.44) |
| Latin America & Caribbean - WB | 90731 (55955-128247) | 76.54 (47.63-108.12) | 182771 (105742-268005) | 62.45 (36.87-90.88) | -0.65 (-0.85--0.45) |
| Middle East & North Africa - WB | 117854 (72453-169708) | 72.27 (44.5-104.7) | 243503 (142512-353017) | 61.25 (36.23-88.95) | -0.48 (-0.69--0.28) |
| North Africa and Middle East | 47229 (16904-84362) | 12.84 (4.5-22.93) | 34082 (7294-70347) | 4.99 (1.02-10.44) | -3.42 (-3.54--3.3) |
| North America | 190 (18-512) | 7.79 (0.83-19.82) | 406 (35-1089) | 7.36 (0.84-18.4) | -0.24 (-0.34--0.15) |
| Oceania | 126752 (58275-204266) | 20.92 (9.71-33.68) | 135763 (60835-231778) | 10.45 (4.66-17.95) | -2.49 (-2.54--2.44) |
| Region of the Americas | 319230 (188857-477789) | 52.22 (30.64-78.3) | 696405 (418131-1019330) | 44.15 (26.78-63.94) | -0.67 (-0.86--0.48) |
| South-East Asia Region | 279764 (169386-416944) | 56.56 (34.09-85.43) | 597872 (378070-852219) | 46.62 (30.29-66.02) | -0.85 (-1.07--0.62) |
| South Asia | 290829 (178032-430632) | 56.79 (34.18-85.68) | 625179 (397730-886358) | 47.29 (30.97-66.38) | -0.8 (-1.02--0.57) |
| South Asia - WB | 75611 (31047-127336) | 32.27 (13.59-55.02) | 183188 (76075-307330) | 33.55 (14.23-56.17) | 0.31 (0.13-0.5) |
| Southeast Asia | 4261 (286-9788) | 9.38 (0.61-21.68) | 4161 (617-9392) | 4.86 (0.71-10.99) | -2.46 (-2.56--2.35) |
| Southern Latin America | 5418 (2092-9373) | 20.96 (8.26-35.97) | 11116 (4344-19204) | 22.26 (8.98-38.56) | 0.3 (-0.17-0.78) |
| Southern Sub-Saharan Africa | 65024 (32103-103053) | 32.04 (16.4-50.4) | 127412 (63919-198985) | 31.38 (16.44-48.82) | -0.06 (-0.26-0.13) |
| Sub-Saharan Africa - WB | 38482 (18548-59929) | 45.47 (22.29-71.18) | 39352 (18127-64826) | 16.98 (7.84-27.93) | -3.4 (-3.45--3.36) |
| Tropical Latin America | 98869 (34181-184112) | 16.3 (5.58-30.37) | 53401 (16273-105461) | 4.89 (1.42-9.72) | -4.5 (-4.65--4.35) |
| Western Europe | 580945 (339464-852181) | 51.3 (29.68-76.84) | 1213841 (699869-1787334) | 44.85 (25.63-66.13) | -0.39 (-0.55--0.23) |
| Western Pacific Region | 19341 (8012-33572) | 23.59 (9.89-40.34) | 36417 (16868-60444) | 22.54 (10.85-36.72) | -0.21 (-0.37--0.05) |
| Western Sub-Saharan Africa | 209792 (67617-396910) | 16.04 (5.16-30.37) | 139780 (37258-284916) | 5.52 (1.4-11.34) | -3.97 (-4.11--3.83) |
| World Bank High Income | 54901 (29576-85751) | 41.04 (22.45-63.26) | 116781 (66399-176448) | 42.44 (24.41-64.06) | 0.16 (0.01-0.32) |
| World Bank Low Income | 439387 (250595-668966) | 46.68 (26.63-71.7) | 952109 (555757-1411488) | 44.59 (26.35-65.41) | -0.22 (-0.42--0.02) |
| World Bank Lower Middle Income | 720486 (401007-1100489) | 49.36 (27.49-76.67) | 1391604 (781836-2099611) | 42.65 (23.93-64.45) | -0.5 (-0.69--0.31) |
