# Supplementary figures and images for: The global burden of lead exposure-related ischemic stroke: based on Bayesian age-period-cohort analysis
Source: Front Public Health. 2025 Jul 16;13:1608129. doi: 10.3389/fpubh.2025.1608129 (PMC12307299; doi:10.3389/fpubh.2025.1608129)

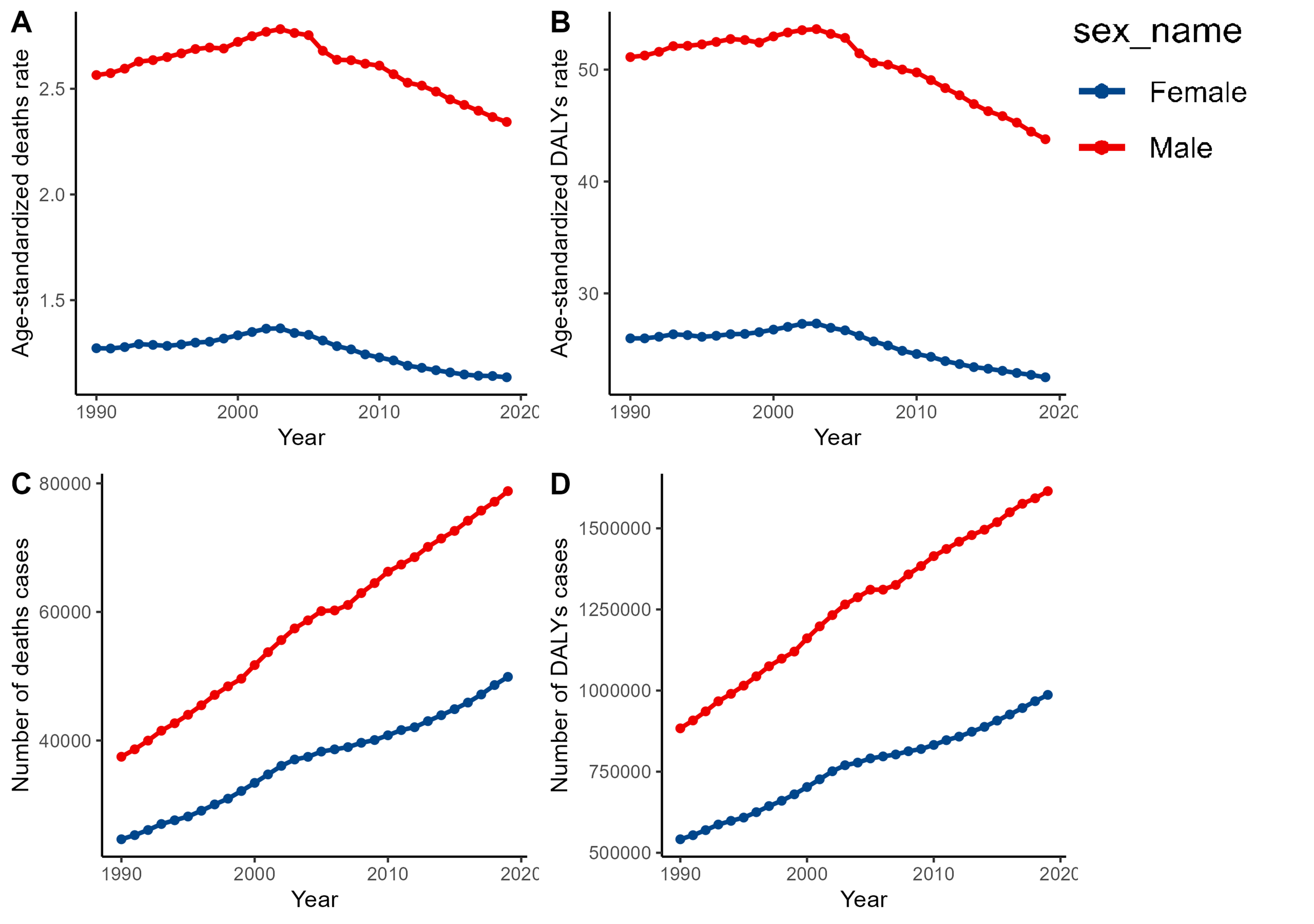

Supplement: Supplementary file 2 [file Image_1.png]

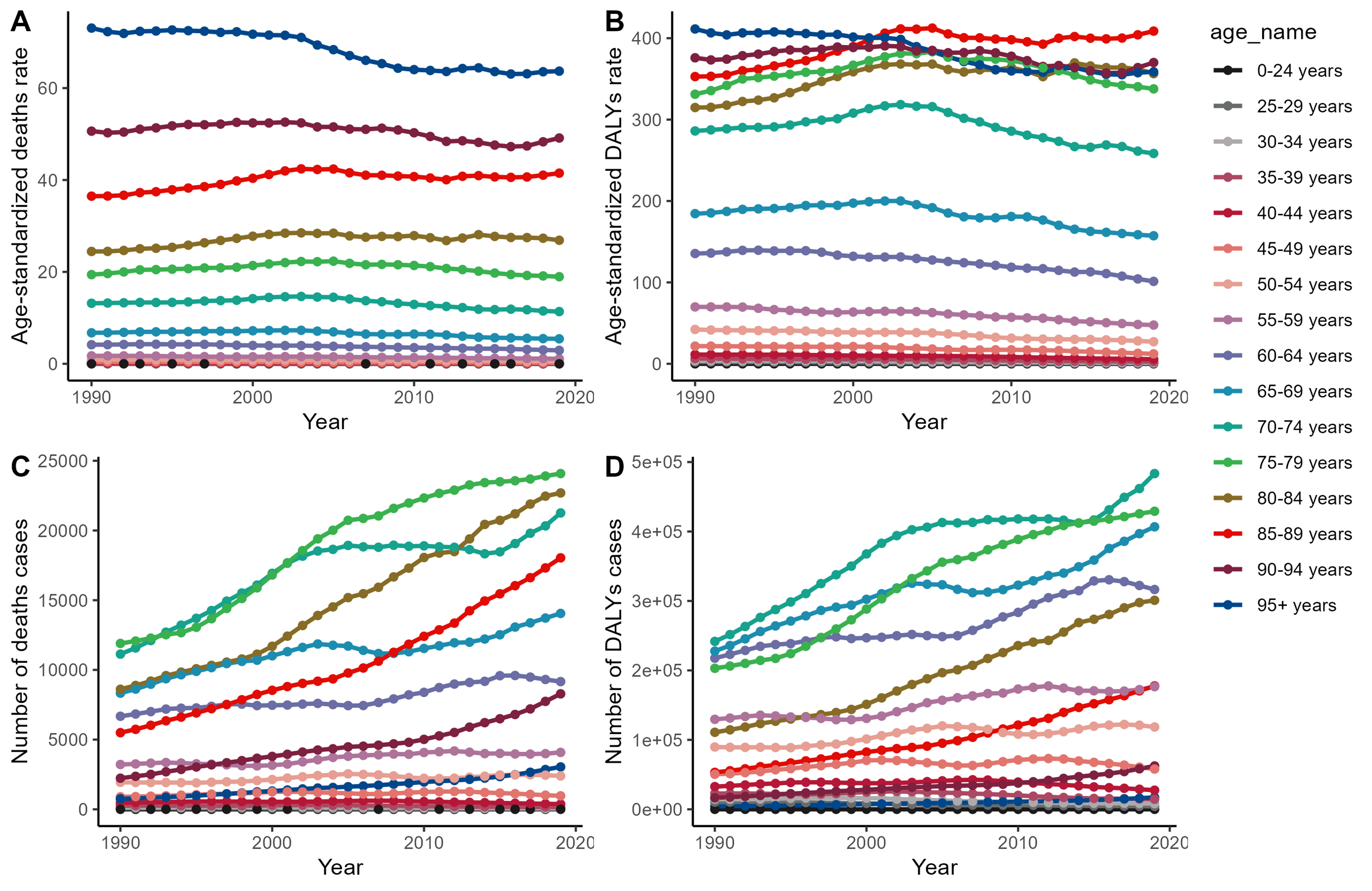

Supplement: Supplementary file 3 [file Image_2.png]

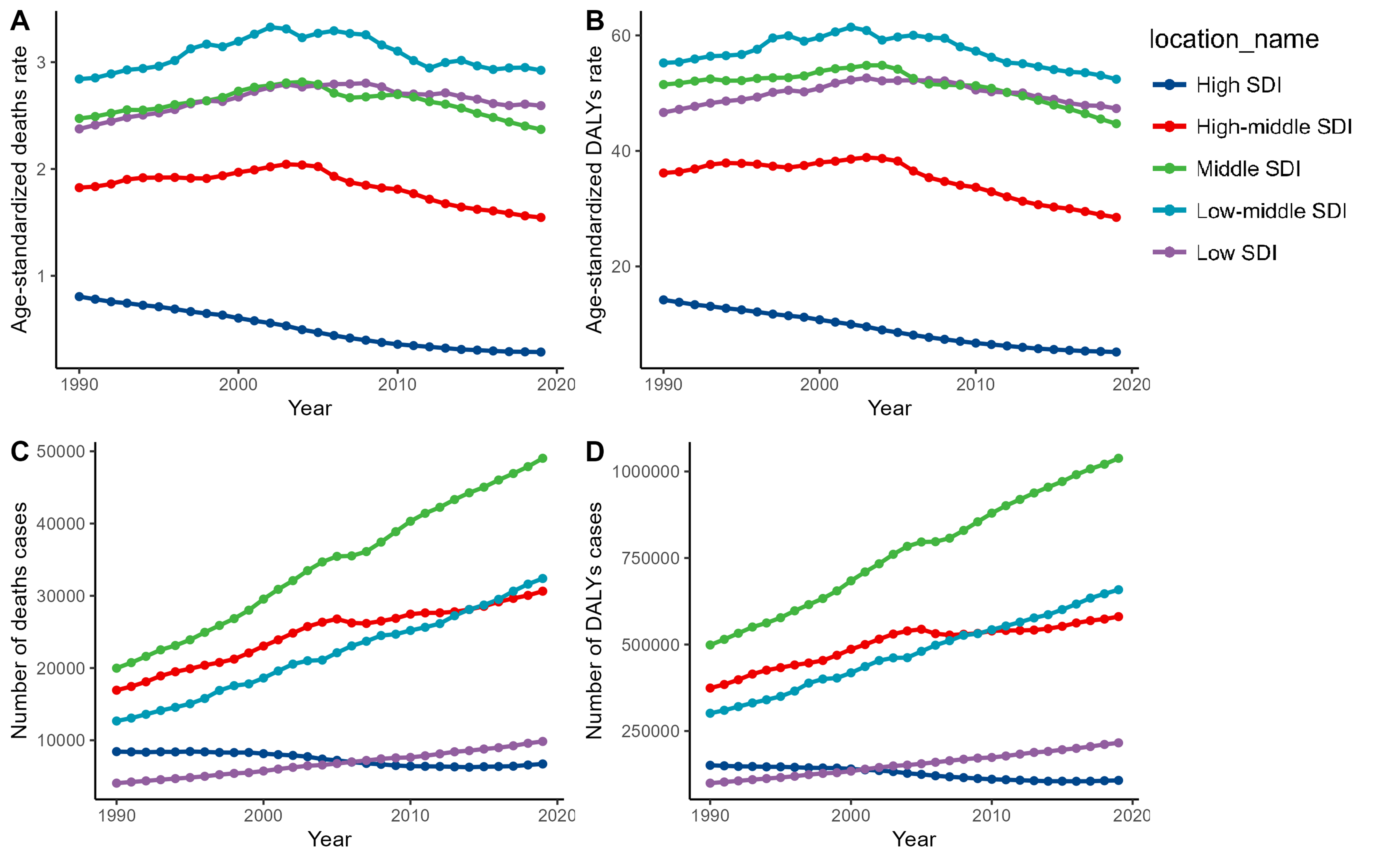

Supplement: Supplementary file 4 [file Image_3.png]
